# Telomere Length and Clonal Hematopoiesis of Indeterminate Potential: A Loop Between Two Key Players in Aortic Valve Disease?

**DOI:** 10.3390/jcdd12040135

**Published:** 2025-04-03

**Authors:** Ilenia Foffa, Augusto Esposito, Ludovica Simonini, Sergio Berti, Cecilia Vecoli

**Affiliations:** 1 CNR Institute of Clinical Physiology, 54100 Massa, Italy; ilenia.foffa@cnr.it; 2 Cardiology Unit, Fondazione Toscana Gabriele Monasterio, 54100 Massa, Italy; aesposito@ftgm.it (A.E.); berti@ftgm.it (S.B.); 3 Department of Surgical, Medical and Molecular Pathology and Critical Area, University of Pisa, 56126 Pisa, Italy; ludovicasimonini@cnr.it

**Keywords:** telomere length, clonal hematopoiesis of indeterminate potential, CHIP, aging, aortic valve stenosis

## Abstract

Aortic valve stenosis (AVS) is the most common valvular heart disease that was considered, for a long time, a passive degenerative disease due to physiological aging. More recently, it has been recognized as an active, modifiable disease in which many cellular processes are involved. Nevertheless, since aging remains the major risk factor for AVS, a field of research has focused on the role of early (biological) aging and its dependent pathways in the initiation and progression of AVS. Telomeres are regions at the ends of chromosomes that are critical for maintaining genome stability in eukaryotic cells. Telomeres are the hallmarks and molecular drivers of aging and age-related degenerative pathologies. Clonal hematopoiesis of indeterminate potential (CHIP), a condition caused by somatic mutations of leukemia-associated genes in individuals without hematologic abnormalities or clonal disorders, has been reported to be associated with aging. CHIP represents a new and independent risk factor in cardiovascular diseases, including AVS. Interestingly, evidence suggests a causal link between telomere biology and CHIP in several pathological disorders. In this review, we discussed the current knowledge of telomere biology and CHIP as possible mechanisms of aortic valve degeneration. We speculated on how a better understanding of the complex relationship between telomere and CHIP might provide great potential for an early diagnosis and for developing novel medical therapies to reduce the constant increasing health burden of AVS.

## 1. Introduction

Aortic valve stenosis (AVS) is the most common valvular heart disease, affecting >25% of subjects over the age of 65 years, with an incidence that increases with age [1]. The predicted exponential growth of the elderly population by 2050 is expected to further amplify the impact of AVS [2,3,4]. Patients with symptomatic AVS have an average life expectancy of less than two years without valve replacement [5], and three-quarters of these patients develop heart failure, undergo valve replacement, or die within five years [6]. No effective medical therapy is available so far. The only treatments able to alleviate symptoms and improve outcomes are surgical or transcatheter aortic valve replacement (SAVR or TAVR) [7,8]. Recent clinical trials also support the beneficial effect of early surgery in patients with asymptomatic, but not critical, aortic stenosis [9,10]. As a result, the use of these procedures has grown exponentially, creating significant clinical and economic challenges. Since it is estimated that the worldwide calcific AVS rates will rise by 240% by 2040 [11], SAVR or TAVR will be unable to meet this growing need. Thus, it is imperative to find an effective medical strategy aimed at early tailored and targeted intervention that is able to prevent or slow disease progression.

The absence of pharmacological treatments is mainly due to a limited knowledge of the molecular mechanisms that drive valvular degeneration and disease progression. For a long time, AVS has been considered as a passive, age-related degenerative disease.

However, more recent studies, linking AVS to modifiable risk factors for coronary artery disease, such as hypercholesterolemia, diabetes, hypertension, smoking, and obesity, have redefined it as an active and potentially modifiable disease [4]. Unfortunately, unlike vascular atherosclerosis, modification of these factors does not significantly improve outcomes in AVS disease. For example, while statins are beneficial in managing atherosclerosis, they have not shown any significant advantages in patients with aortic stenosis [12]. Moreover, coexisting disorders, i.e., end-stage renal disease, are risk factors for AS [8]. The use of some supplements, such as vitamin D or calcium, might influence the process of AVS, although data have been inconsistent [8].

Valvular heart disease affects approximately 1–2% of young individuals who engage in regular exercise within the general population [13]. Although data are limited, there is a theoretical possibility that a high stroke volume, combined with vigorous mechanical contractions of the heart and an exercise-induced increase in chronotropic activity, may accelerate valve dysfunction. Notably, calcific aortic stenosis is frequently observed in master’s athletes, a population that maintains high levels of physical activity well into old age. Interestingly, also in this case, while sustained physical activity has a beneficial impact on various risk factors for atherosclerotic heart disease—such as plasma lipoprotein levels, systolic blood pressure, and glucose metabolism—it does not appear to reduce the risk of developing valvular heart disease [13].

Aortic valve degeneration is characterized by a cascade of molecular derangements, starting with fibrotic thickening and progressing to the extensive calcification of the valve leaflets. Many cellular processes are involved, from endothelial dysfunction to inflammation, lipid deposition, matrix remodeling, and calcification [14,15,16,17]. Despite these findings, aging remains the primary risk factor for AVS, suggesting that early biological aging may play a critical role [4].

It is well established that individuals age at different rates. Indeed, biological aging (also defined as functional or physiological aging), which reflects the real physiological state of an organism as a result of genetic background and environmental stressors (including inflammation, oxidative stress, and lifestyle choices) is often different from chronological aging, which simply denotes the passing of time. Thus, whether chronological aging alone may not directly cause aortic valve degeneration (as many elderly individuals have no AVS), all those factors underlying biological aging may play a key role.

One of the central mechanisms of aging with a well-recognized role in cardiovascular disease involves the changes in the length of telomeres (telomere length, TL), specialized structures located at the ends of eukaryote chromosomes. Telomere length has been classified as a trigger and/or an amplifier of the molecular pathways driving the age-related diseases [18]. Clonal hematopoiesis of indeterminate potential (CHIP), a pathological mechanism closely associated with aging, has emerged as a novel and independent risk factor for cardiovascular diseases [19]. A causal link between telomere biology and CHIP across various conditions, including cardiovascular disease, is emerging [20].

In this review, we discussed the current knowledge of telomere biology and CHIP as potential pathological mechanisms contributing to aortic valve disease. Finally, we speculated on how a deeper understanding of the interplay between telomeres and CHIP could offer great potential for the development of effective medical therapies, ultimately helping to reduce the growing health burden of aortic valve stenosis.

## 2. Structure and Cells of the Aortic Valve

The aortic valve is a complex structure that is able to open with low impedance to unidirectional flow and close with enough strength to bear systemic blood pressure loading at each cardiac cycle. The valve leaflet is composed of three layers with distinct properties [21,22]: the lamina ventricularis, which faces the left ventricle, is rich in radially oriented collagen and elastin to ensure elasticity and recoil and is innervated by sympathetic fibers [23]; the spongiosa layer, in the middle, contains glycosaminoglycans and proteoglycans to ensure lubrication between the two other layers; the fibrosa consists of circumferentially aligned collagen fibers. The valve leaflets are mainly avascular and receive oxygen and nutrients via passive diffusion from the circulating blood.

Two main cell types inhabit a healthy aortic valve: the valvular interstitial cells (VICs) and the valvular endothelial cells (VECs), both mechanosensitive and responsive to stretch, compression, and blood shear forces [24]. The VICs, the most abundant, are involved—at least in healthy conditions—in the maintenance of the extracellular matrix [25] but, upon pathological signals, they can undergo osteogenic differentiation [26].

The VECs form a monolayer that covers both the fibrosa and the ventricularis sides of the valve leaflets. They are phenotypically distinct from vascular endothelial cells and differently oriented. Indeed, while vascular endothelial cells are oriented parallelly to the direction of blood flow, the VECs are perpendicularly oriented [27,28]. Moreover, the VECs of ventricularis are exposed to a linear shear stress, while those of the fibrosa are exposed to flow vortices within the sinuses of Valsalva and, thus, subjected to oscillatory low shear stress, which is able to promote calcification [29,30,31,32].

## 3. The Two Phases of Aortic Valve Calcification

Sclerosis and stenosis are progressive, sequential stages of aortic valve calcification. Endothelial dysfunction of the fibrosa layer is the main trigger of valve degeneration and is governed by inflammation, subendothelial lipoprotein oxidation, fibrosis, and microcalcification, while the later phases are governed by a self-perpetuating cycle of gradual calcification [4].

Under physiological conditions, the mechanical stress exerted by blood flow over time can contribute to the onset of aortic valve sclerosis [33], as VECs are subjected to oscillatory low shear stress, which promotes sclerosis during systole, and turbulent flow vortices during diastole. While physiological shear stress alone does not directly lead to aortic valve degeneration—evidenced by the significant number of elderly individuals without AVS [34]—genetic and non-genetic factors play a key role in triggering the degenerative process [34]. For example, the congenital bicuspid aortic valve (BAV) phenotype results in altered mechanical stress, which can induce the early onset of aortic valve degeneration and accelerate disease progression, with the dilation of ascending aorta [35]. Consequently, individuals with BAV are nearly 25 times more likely to develop severe AVS compared to those with a tricuspid valve [36,37].

Endothelial dysfunction leads to lipid deposition from the bloodstream into the subendothelial space. This process allows inflammatory cells and cytokines to infiltrate the valvular interstitial space, promoting endothelial-to-mesenchymal transition (EndMT), which ends in a novel myofibroblastic cell phenotype migrating into the interstitial space [14,15,38]. The role of the activated myofibroblast-like VECs in the definition of the extracellular matrix is not completely known [14], although it is conceivable that this transformation alters a number of molecular pathways that ensure valvular tissue homeostasis, such as the production of nitric oxide (NO) [39,40].

Valvular endothelial NO plays pivotal roles in maintaining valve physiology by inhibiting fibrosis and calcification [39,40]. It also enhances NOTCH1 signaling in the VICs, which, in turn, suppresses osteoblast-related regulators such as RUNX2 and promotes the expression of anti-calcification factors [41]. Thus, valvular endothelial cell dysfunction and the subsequent reduced NO production promote a fibrotic process within the valve. The VECs under increased shear stress promote the activation of quiescent VICs in the three layers of the valve, driving their differentiation into myofibroblasts. The myofibroblastic VICs secret structural matrix proteins and matrix metalloproteinases, contributing to extracellular matrix remodeling, leaflet thickening, and increased stiffness [26]. Microcalcification, in the early phase, may rise from the death of myofibroblastic VICs, which release apoptotic bodies in areas of lipid deposition and inflammation [16].

Besides apoptosis, other processes of cell death play a crucial role in valve calcification, including autophagic cells that release vesicles able to attract inflammatory cells [42]. Of note, in specific conditions (i.e., inorganic phosphate levels), autophagy might serve as a survival mechanism in VICs to guarantee protection from calcification [43,44]. Furthermore, Bonetti et al. observed a release of pro-calcific lipid material (primarily acidic phospholipids) during VIC mineralization dependent on massive cell membrane dissolution caused by the overexpression of a specific phospholipase (cPLA2α) [45]. On the other hand, it is fundamental to note that VICs exhibit remarkable plasticity, with some differentiating into secretory myofibroblasts in response to injury or stress. However, VICs can also undergo pro-calcific degeneration, irrespective of their prior differentiation state, particularly under conditions of chronic inflammation or dysregulated mineral metabolism [46].

The mechanisms of aortic valve calcification are not totally known, although there is accumulating evidence that VECs and VICs interact to ensure the proper development and maintenance of the aortic valve. Indeed, under conditions of physiological shear stress, for instance, the VEC protects the VIC from myofibroblastic differentiation (by producing NO) [41,42,43,44,45,46,47], while the VIC can suppress EndMT and the osteogenic differentiation of VEC [48]. A disruption of the interaction between VICs and VECs could probably contribute to valve pathology and subsequent calcification.

**Figure 1:**
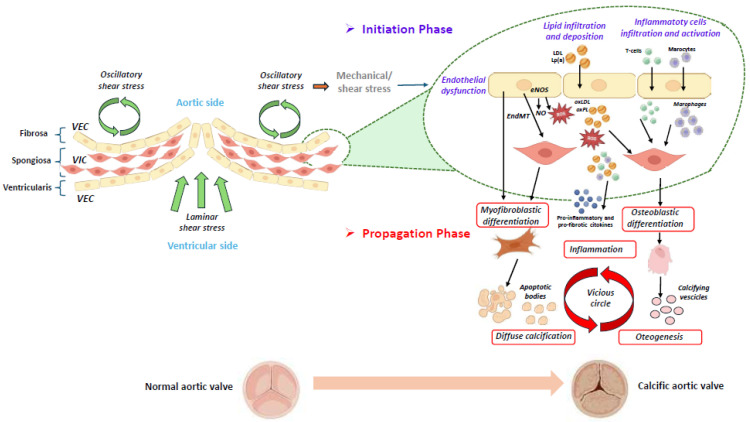
Schematic representation of the initiation and progression phases of calcific aortic valve disease [4,14,16,17]. The aortic valve consists of three layers: the ventricularis, the spongiosa, and the fibrosa. A monolayer of valvular endothelial cells (VECs) covers the surface of the aortic valve, and valvular interstitial cells (VICs) are distributed throughout the three layers of the aortic valve. The initiation and progression of aortic valve calcification are two distinct phases, in which different molecular pathways are involved. In the first phase, different stimuli, such as mechanical/shear stress induce the endothelial dysfunction of VECs, which allows lipid infiltration and deposition (i.e., LDL (low-density lipoprotein) and Lp(a) (lipoprotein[a]), accompanied by the infiltration and activation of inflammatory cells. A dysregulation of the eNOS (endothelial nitric oxide synthase) pathway induces the production of reactive oxygen species (ROS), which stimulates the oxidation of infiltrated lipids into ox-LDL (oxidized LDL) and oxidized phospholipids (ox-PLs). These molecules are further able to promote the apoptosis of the valvular interstitial cells (VICs) and the release of apoptotic bodies, leading to diffuse calcification. Following the oxidation of lipids in the valve, the immune cells infiltrate the tissue and are activated. In the second phase, inflammation, together with osteogenic differentiation and calcification, is involved in a vicious circle, which is responsible for disease progression. (The illustration was created using BioRender, https://www.biorender.com/, accessed on 29 January 2025).

As the disease progresses, the continuous remodeling of the extracellular matrix and ongoing calcification impair leaflet motion, leading to altered mechanical stress. This stress further perpetuates a vicious cycle of chronic inflammation and calcification [14,16] (Figure 1).

## 4. Telomere Length in Aortic Valve Calcification

Cellular senescence is a universal cellular response to internal and/or external stressors, in which a cell remains metabolically active, but permanently exits the cell cycle, becoming unresponsive to proliferation-inducing signals. This phenomenon was first described by Hayflick L. and Moorehead P., who showed that cultured cells could only undergo a limited number of divisions before entering an irreversible cell cycle, a process called replicative senescence [49]. Further studies demonstrated that this limitation is partially due to signals derived from the shortening of telomeres—specialized structures localized at the ends of chromosomes—which occurs, physiologically, during each DNA duplication [50,51].

Telomeres consist of highly repetitive DNA sequences (in humans, TTAGGG) and a six-protein complex called shelterin. Telomeres serve as protective caps that are essential for maintaining genomic stability. Their length is maintained by the holoenzyme telomerase, a ribonucleoprotein with an RNA subunit (TERC) and a reverse transcriptase enzymatic subunit (TERT). Except for a few cell types (i.e., germ cells), most human cells, including leukocytes, lack telomerase activity and therefore are not able to maintain the telomere length. Therefore, telomeres gradually shorten (25–200 bases) in each replication cycle [52]. As a result, in humans, their length is inversely correlated with age, and once telomeres reach a critical length (becoming excessively short)—the “Hayflick limit”—the cell enters replicative senescence [53]. Approximately 8.1% of TL variation is genetically determined, while the remaining variation is largely explained by environmental factors and cumulative stress [54,55]. Thus, leucocyte TL (LTL) reflects exogenous environmental exposures that cause genotoxic damage to the DNA of hematopoietic stem cells, as well as endogenous pathways related to biological aging. Hence, LTL serves as a biomarker of genomic instability, integrating the cumulative effects of biological stress over time. Immune cells can remove age-related, replicative senescent cells in order to prevent their accumulation, but they become powerless under conditions of heightened stress or immune dysfunction, [56]. This accumulation contributes to chronic inflammation, organ dysfunction, and disease progression [56,57,58].

Senescent cells are metabolically active cells that secrete several factors, including cytokines, chemokines, and matrix metalloproteinases, collectively known as senescence-associated secretory phenotype (SASP). SASP contributes to extracellular matrix remodeling and valvular structural changes associated with aging [56].

Inflammation, oxidative stress, and hemodynamic stress can accelerate TL shortening and induce premature cellular senescence [59]. Over time, this creates a vicious cycle, where pathological senescent cells accumulate, exacerbating tissue degeneration through their secretory activity and by inducing senescence in neighboring cells (bystander effect) [59,60,61,62]. Slowing or eliminating cellular senescence might represent an innovative therapeutic strategy for all aging-dependent diseases, including aortic valve stenosis. Unfortunately, human applications of these potential therapies are still limited by the limited knowledge of the basic molecular cell biology of senescence.

A great number of clinical and epidemiological studies have focused on the role of TL in cardiovascular disease, especially atherosclerosis. Evidence highlights that a short leukocyte TL is associated with atherosclerotic disease and increased cardiovascular mortality [62,63,64,65]. Data about the implication of TL in aortic valve calcification are scarcer and more controversial. Kurz et al. (2006), in a population of older individuals (≥70 years) reported that calcific aortic stenosis, but not coronary disease, was associated with shorter TL [66]. More recently, Saraieva et al. (2021) observed that TL was shorter in calcified regions of aortic valves compared to non-calcified areas in AVS patients [67]. Additionally, TL in non-calcified areas of valves from AVS patients was shorter than in valves from control subjects, suggesting that localized telomere shortening may precede aortic valve calcification and contribute to AVS risk [67]. This finding is in line with the results obtained in an experimental study in *Notch1* haploinsufficient mice (*Notch1^+/−^ mTRG1–3*), in which telomere shortening elicited age-dependent tricuspid AVS and aortic valve calcification via the RUNX2 pathway [68].

Oxidative stress and chronic inflammation may be the possible common denominator for TL shortening and early valve degeneration, since they may cause local telomere attrition and valve calcification [69,70]. Likewise, short telomeres in calcified areas can partially reflect an increased presence of immune cells that largely infiltrate lesions [71].

TL-associated single nucleotide polymorphism studies have shown that alleles associated with shorter TL are overrepresented in individuals with clinical manifestations of atherosclerotic cardiovascular disease [72,73], assuming a causal role of short TL in atherosclerotic development. Actually, traditional observational studies are unable to provide an effective, causal relationship between exposure and outcome, since they are susceptible to confounding factors or reverse causality [74,75]. Conversely, Mendelian randomization (MR] is now a valuable method for revealing a causal link between an exposure and an outcome, using genetic variants as instrumental variables [74,76]. Recently, Wang et al. (2022) conducted the first large-scale MR analysis using genome-wide association data to explore the relationship between TL and calcific AVS [77]. Their work strongly suggests that telomere biology, especially inherited short TL, is potentially involved in the development of AVS.

In peripheral blood cells, short telomere length reflects two processes in the leukocytes. First, short LTL can represent an age-dependent decline, triggered by a loss of TL in stem and progenitor cells. Secondly, it can denote a shift in leukocytes from subpopulations with long telomeres (myeloid cells] to those with shorter telomeres (memory lymphocytes] [78]. Therefore, in the hematopoietic system, if telomere shortening may lead to proliferative impairment and cellular senescence, longer telomeres maintain cell division activity and the inflammatory responsiveness of immune cells. Interestingly, in a large study of patients undergoing TAVR, Hoffmann et al. (2022) found that longer a LTL was associated with increased mortality after valve replacement [79]. This result appears to be in contrast with the findings by Steinmetz et al. (2019) in a similar population of elderly patients undergoing TAVR [80]. To explain the unexpected result, Hoffmann et al. (2022) proposed the “inflammatory potential hypothesis”, suggesting that longer telomeres might enhance myeloid inflammation and increase adverse outcomes in this specific old (≥70–80 years) population [79,81]. Indeed, despite the success of valve replacement, a considerable number of patients with an inflammatory background remain at risk of adverse outcomes. Murine studies have shown that very short telomeres may protect against atherosclerosis by limiting myeloid cell proliferation, a hallmark of this inflammatory disease [82]. Hoffmann et al. also reported that patients with longer TL had higher neutrophil and monocyte levels, as well as increased inflammatory markers, indicating that TL may serve as a surrogate for increased myeloid inflammation in particular patient groups [79]. This finding highlights the complexity of TL’s role in cardiovascular disease and underscores the need for further research.

## 5. Clonal Hematopoiesis of Indeterminate Potential in Aortic Stenosis

Hematopoietic stem cells (HSCs) are responsible for the generation of all mature blood cells throughout life. With age, genomic instability and acquired somatic mutations of HSCs gradually occur, and some clones disproportionately occupy the bone marrow and peripheral blood cells. Clonal hematopoiesis (CH) arises when a single HSC-derived lineage dominates the production of mature blood cells. This clonal expansion of mutated cells has been linked to an increased risk of hematologic diseases [83]. Individuals with expanded blood cell clones but without hematologic malignancies are considered to have CHIP.

CHIP is defined as somatic mutations of a leukemia-associated gene with variant allele frequency (VAF) ≥ 2%, normal peripheral blood counts, and no clinical or pathological evidence of hematologic malignancy or other clonal disorders [84]. CHIP is rare in individuals under 40 years but is present in over 10% of those aged 70 or older [85]. People with CHIP have a high risk of all-cause mortality, mainly due to an increased risk of cardiovascular disease [85,86]. The exact mechanism that links CHIP and CVD remains unclear but is thought to involve chronic inflammation [4] induced by aging, also defined as “inflammaging” [87]. Indeed, many mutations causing clonal expansion of blood stem cell clones also promote increased expression of inflammatory genes in innate immune cells [86,88,89].

Age-related clonal hematopoiesis was first identified approximately 25 years ago through studies on X-chromosome inactivation patterns [90,91,92]. However, only the development of next generation sequencing technologies has allowed for the study of CHIP frequency, as well as its longitudinal clinical consequences and gene-specific characterization in a large number of individuals.

Clinical studies in patients with cardiovascular disease have identified *DNMT3A*, *TET2*, and *ASXL1* genes as the most commonly mutated CHIP-driver genes, accounting for ~80% of all CHIP cases [86,87,88,89,90,91,92,93]. Other mutations have been found in *JAK2* gene and are associated with increased risk of thrombosis, DNA damage response pathway genes *PPM1D* and *TP53*, and mRNA splicing factors *SRSF2* and *SF3B1*. Mutations in these genes are commonly observed in hematologic malignancies, including myelodysplastic syndrome, myeloproliferative neoplasms, and acute myeloid leukemia [93].

The first clinical study in patients with AVS was performed by Mas-Peiro et al. in 2020 and showed that *DNMT3A*- or *TET2*-CHIP-driver mutations occurred frequently in patients with severe AVS [94]. The frequency of these mutations increased with age and was associated with a threefold higher mortality risk during the first 8 months after TAVI (by approximately 3-fold).

Since in murine models of cardiac disease *Tet2*- and *Dnmt3A*-loss of function activated the inflammasome complex [86] and promoted fibrosis development [95], it is conceivable that *Dnmt3A* or *Tet2*-CHIP-driver mutations contributed to the development of AVS.

Both *DNMT3A* and *TET2* gene mutations increase the inflammatory states but via different mechanisms. Indeed, patients with a *DNMT3A* mutation have a significantly higher ratio of the proinflammatory Th17 cells to the anti-inflammatory regulatory T cells, while patients with a *TET2* mutation showed increased levels of CD14dimCD16++ monocytes, which are able to release high levels of proinflammatory cytokines (including TNFα, IL-1β, and IL-8). Individuals who carry *DNMT3A* or *TET2* CHIP-driver sequence variations displayed increased expression of proinflammatory cytokines, IL-6, and cellular receptor CD163, as well as the NLRP3 inflammasome complex and other genes involved in cytokine release syndrome [96].

Preliminary data from the Vanderbilt biobank (BioVU) showed that patients with CHIP have almost twice the risk of developing degenerative aortic stenosis [97]. Recently, an NGS analysis of 67 genes was performed in 258 patients with aortic valve stenosis undergoing TAVR to test their association with long-term survival after TAVR [98]. Somatic variants driving clonal hematopoiesis were highly prevalent in this cohort of AVS patients, with a CHIP-positive rate of 68% (patients presenting at least 1 CHIP-driver mutation with a VAF ≥ 2%). In line with previous findings, *DNMT3A* and *TET2* were the most frequently mutated genes, and the prevalence of CHIP increased with age. Acquired variants in *TET2* were strongly associated with poor long-term survival after TAVR; conversely, AVS patients carrying mutations in *DNMT3A* had a survival rate similar to those without CHIP genetic variants. This finding might suggest a “protective effect” of *DNMT3A* mutations (at least at low variant allele frequencies, 2–10%) with respect to mortality after TAVR. Patients who carried both *DNMT3A* and *TET2* mutations exhibited slightly improved post-TAVR survival as compared with individuals without CHIP. The mechanisms by which *TET2*-mediated CHIP promotes disease probably have a greater effect on post-TAVR mortality compared to *DNMT3A*-mediated CHIP. However, it may be plausible that larger *DNMT3A* clones (as a result of a different VAF cut-off) are less benign after TAVR. Other analyses in larger patient cohorts are required to confirm this association; however, the presence of *TET2* mutations in the blood may represent a novel prognostic indicator for poor survival after TAVR and guide the clinical care of patients with these mutations [99].

Finally, Kim M et al. (2024), in a prospective case-control study, performed a NGS analysis of 24 genes (*ASXL1*, *BCOR*, *CALR*, *CEBPA*, *DNMT3A*, *ETV6*, *FLT3*, *IDH1*, *IDH2*, *JAK2*, *KIT*, *KMT2D*, *KRAS*, *MPL*, *NPM1*, *NRAS*, *RUNX1*, *SETD2*, *SF3B1*, *STAG2*, *TET2*, *TP53*, *U2AF1*, and *WT1*) associated with CHIP variants and cardiovascular diseases [100]. The study clearly proved a higher proportion of CHIP variants in individuals with AVS compared to age- and sex-matched controls. Interestingly, their study also reported that the major CHIP variants detected were in *DNMT3A* and *TET2* genes [100].

Since mutations in different CH-driver genes generate distinct pro-inflammatory profiles that are able to trigger different molecular mechanisms, the scientific community will need to make further efforts to characterize the cellular pathways by which the various CH-driver gene mutations contribute to aortic valve degeneration. Interventions to counteract the effect of CHIP variants, including CHIP-mediated inflammation, will allow for improving early AVS diagnosis and medical treatment.

## 6. Future Directions: A Possible Causal Link Between TL and CHIP in AVS

The complex, bidirectional relationship between LTL and CHIP is supported by several studies. Nevertheless, while cross-sectional analyses showed a correlation between CHIP and shorter-measured LTL (independently of age) [101], in genome-wide association analyses, CHIP was associated with longer-measured LTL [54]. Whether and how CHIP and LTL are causally associated is still unknown.

Recently, Nakao et al. (2022) have investigated the relationships between LTL, CHIP, and coronary artery disease using measured LTL (mLTL) and genetically imputed LTL (gLTL) data from the National Heart, Lung, and Blood Institute (NHLBI) Trans-Omics for Precision Medicine (TOPMed) program and the UK Biobank [102]. Through bidirectional Mendelian randomization analysis, the researchers demonstrated that the processes promoting LTL elongation initially increase the risk of CHIP development. CHIP subsequently accelerates LTL shortening [102].

Genetic association studies have shown that the *TERT* locus is the strongest genetic region for CHIP susceptibility, which also impacts telomere length [20]. This double association with the *TERT* locus greatly supports a possible causal relationship between telomere biology and CHIP. Kar et al. (2022) also indicated a causative link between telomere length and CHIP using mendelian randomization analyses [103]. These authors suggested that longer LTL and smoking were causal risk factors for CH. Moreover, genetic predisposition to CH increases the risks of myeloproliferative neoplasia, nonhematological malignancies, atrial fibrillation, and blood epigenetic aging.

Very recently, a systematic characterization of rare variant associations with LTL through an exome-wide association study (ExWAS) among 390,231 individuals in the UK Biobank allowed for the identification of 18 rare-variant genes for LTL. This study identified three novel genes (*ASXL1*, *CFAP58*, and *TET2*) associated with LTL. Curiously, *ASXL* and *TET2* genes are strictly associated with CHIP [104].

Among the other genes known to also be implicated in monogenic telomere disorders, *POT1*, which encodes the subunit of the telomeric structure, has been recently found to be associated with CHIP by DeBoy et al. (2023) [105]. Specifically, *POT1* mutations were associated with long telomere length and conferred a predisposition to a familial clonal hematopoiesis syndrome associated with benign and malignant solid neoplasms [105]. The risk of these phenotypes was mediated by extended cellular longevity and by the capacity to maintain telomeres over time [105].

**Figure 2:**
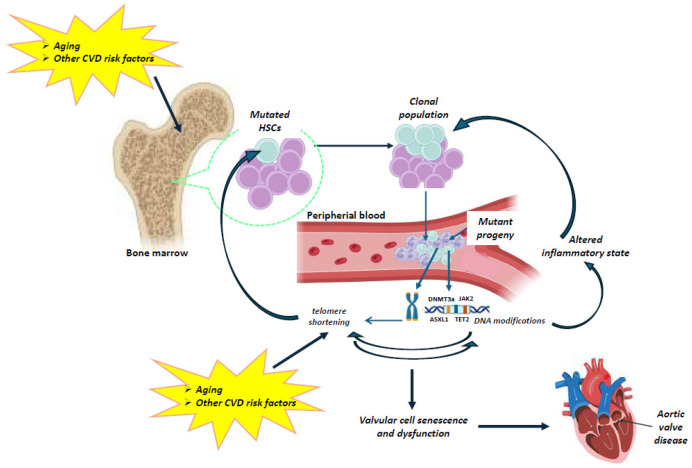
Schematic representation of the possible relationship between telomeres and CHIP in aortic valve disease. Aging results in gradual leukocyte telomere attrition. Telomere shortening and telomerase dysfunction can lead to genomic instability and ultimately to CHIP. Very limited data are available on the interplay between telomere biology and clonal hematopoiesis. However, current clinical evidence may allow us to speculate a direct association between telomere attrition and CHIP. Aging, as well as other cardiovascular risk factors (e.g., smoking, environmental exposure) influence the occurrence of mutations in a CHIP-driver gene of hematopoietic stem cells (HSCs), resulting in the expansion of a mutated HSC clone and mutant circulating leukocytes. Clonal expansion can promote inflammation and intensify telomere shortening in circulating cells, which further accelerates the adverse effects of telomere dysfunction on the valvular cells, contributing to aortic valve disease. (The illustration was created using BioRender, https://www.biorender.com/, accessed on 29 January 2025).

To date, the interplay between telomere biology and clonal hematopoiesis in aortic valve disease remains undefined and underexplored. However, the available data support the notion that telomeres and CHIP may play a causal, bidirectional role in valvular aging and stenosis (Figure 2).

Indeed, on the one hand, aging results in gradual leukocyte telomere attrition, which further leads to genomic instability and ultimately to CHIP. On the other hand, aging, as well as other cardiovascular risk factors (e.g., smoking, environmental exposures) influence the occurrence of a mutation in a CHIP-driver gene in HSCs, resulting in the expansion of a mutated HSC clone and mutant circulating leukocytes. The clonal expansion can promote inflammation, which in turn intensifies telomere shortening in the blood. The telomere dysfunction increases valvular cell senescence and dysfunction, contributing to aortic valve disease. Thus, a combined study of the telomere–CHIP interplay may be of pivotal importance for the development of both prevention strategies and novel medical treatments in calcific aortic stenosis.

## 7. Conclusions

AVS is a complex trait disorder, which reflects interactions between genetic and environmental risk factors, although aging remains the pivotal risk factor. Early valvular heart disease is asymptomatic and progresses slowly [106]. Hence, although early drug intervention might be more effective in preventing disease progression, current clinical practice can identify (and treat) only the later stages of the disease, when calcification is already present. Evidence suggests a causal association of both TL and CHIP with valve degeneration, but a joint, causal role of these two molecular mechanisms has been unexplored so far. Elucidating this bidirectional relationship could be helpful in identifying the first stages of AVS disease, prior to the onset of irreversible macroscopic features of later stages. Moreover, the knowledge of their mutual causal effects could be successful in developing novel personalized medical treatments to prevent or slow the degeneration of aortic valve.

## Data Availability

No new data were created or analyzed in this study. Data sharing is not applicable.
